# In this month’s Bulletin

**DOI:** 10.2471/BLT.18.000218

**Published:** 2018-02-01

This month’s issue has a special focus on emerging infectious diseases and antimicrobial resistance to accompany this year’s Prince Mahidol Award Conference in Thailand. 

 In editorials, Tedros Adhanom Ghebreyesus and Patricia Espinosa (78) draw attention to an initiative to protect people in small island developing states from the health impacts of climate change. Etienne Langlois et al. (79) argue that qualitative evidence should be used to improve health guidelines.

 In the news section, Sophie Cousins (82–83) reports on efforts to control bovine tuberculosis and prevent human exposure to infected meat and dairy products. Marion Koopmans tells Fiona Fleck (84–85) why the world needs a new global network to respond to emerging infectious diseases.

## Zambia

### Heading off an urban cholera outbreak

Marc Poncin et al. (86–93) study the mass use of oral vaccines.

## Thailand

### Antibiotic use in poultry 

Gumphol Wongsuvan et al. (94–100) survey farmers on the quantities of antibiotics used when raising poultry for human consumption.

### Where do the antibiotics go?

Angkana Sommanustweechai et al. (101–109) trace imports, manufacturing, distribution and regulation.

## Global

### From laws to laboratories

Amitabh B Suthar et al. (110–121) review lessons learnt in implementing the International Health Regulations.

### Describing cough and fever

Julia Fitzner et al. (122–128) examine clinical case definitions for influenza-like illness and severe acute respiratory infection.

### Counting economic and human costs

Victoria Y Fan et al. (129–134) quantify expected losses from future pandemics.

### Preventing antimicrobial resistance

Jude Nwokike et al. (135–137) argue for more stringent quality assurance measures.

### Returns on investment in human, animal and ecosystem health 

Daniel L Schar et al. (138–140) present an investment case for the one health approach.

### Addressing supply and demand 

Viroj Tangcharoensathien et al. (141–144) consider interventions for improving antibiotic use. 

